# Dentists’ Awareness, Perception, and Attitude Regarding COVID-19 and Infection Control: Cross-Sectional Study Among Jordanian Dentists

**DOI:** 10.2196/18798

**Published:** 2020-04-09

**Authors:** Yousef Khader, Mohannad Al Nsour, Ola Barakat Al-Batayneh, Rami Saadeh, Haitham Bashier, Mahmoud Alfaqih, Sayer Al-Azzam, Bara’ Abdallah AlShurman

**Affiliations:** 1 Jordan University of Science and Technology Irbid Jordan; 2 Global Health Development/Eastern Mediterranean Public Health Network Amman Jordan

**Keywords:** COVID-19, infection, dentist, infection control

## Abstract

**Background:**

Despite the availability of prevention guidelines and recommendations on infection control, many dental practices lack the minimum requirements for infection control.

**Objective:**

This study aimed to assess the level of awareness, perception, and attitude regarding the coronavirus disease (COVID-19) and infection control among Jordanian dentists.

**Methods:**

The study population consisted of dentists who worked in private clinics, hospitals, and health centers in Jordan. An online questionnaire was sent to a sample of Jordanian dentists in March 2020. The questionnaire was comprised of a series of questions about dentists’ demographic characteristics; their awareness of the incubation period, the symptoms of the disease, mode of transmission of COVID-19 and infection control measures for preventing COVID-19; and their attitude toward treating patients with COVID-19.

**Results:**

This study included a total of 368 dentists aged 22-73 years (mean 32.9 years, SD 10.6 years). A total of 112 (30.4%) dentists had completed a master or residency program in dentistry, 195 (53.0%) had received training in infection control in dentistry, and 28 (7.6%) had attended training or lectures regarding COVID-19. A total of 133 (36.1%) dentists reported that the incubation period is 1-14 days. The majority of dentists were aware of COVID-19 symptoms and ways of identifying patients at risk of having COVID-19, were able to correctly report known modes of transmission, and were aware of measures for preventing COVID-19 transmission in dental clinics. A total of 275 (74.7%) believed that it was necessary to ask patients to sit far from each other, wear masks while in the waiting room, and wash hands before getting in the dental chair to decrease disease transmission.

**Conclusions:**

Jordanian dentists were aware of COVID-19 symptoms, mode of transmission, and infection controls and measures in dental clinics. However, dentists had limited comprehension of the extra precautionary measures that protect the dental staff and other patients from COVID-19. National and international guidelines should be sent by the regional and national dental associations to all registered dentists during a crisis, including the COVID-19 pandemic, to make sure that dentists are well informed and aware of best practices and recommended disease management approaches.

## Introduction

### Background

The coronavirus disease (COVID-19) is a newly discovered viral infection that started in Wuhan, China and caused the outbreak of pneumonia in the rest of the world. It seems that the rapidly spreading virus is more contagious than severe acute respiratory syndrome coronavirus and Middle East respiratory syndrome coronavirus [1]. A suggested route of human-to-human transmission is through airborne droplets, touching or coming into contact with an infected person or a contaminated surface. Moreover, other routes such as blood or saliva have not been explored but are possible because of the documented transmission of blood-borne infectious diseases such as HIV/AIDS, hepatitis C virus, and hepatitis B virus through blood or saliva. These routes of transmission increase the concern about a similar route of transmission for COVID-19 in the dental setting [2].

### COVID-19 and Dental Treatment

A large number of medical staff were reported to have acquired the disease while working with infected individuals [3]. The dental clinic is not an exception for a similar possibility of transmitting and acquiring the infection between staff or individuals; moreover, the dental clinic could be a riskier environment for spreading the virus because of the close contact with patients and the nature of the dental treatment [4]. Although patients diagnosed with COVID-19 are not supposed to receive dental treatments, dental emergencies can occur, and close contact would be unavoidable. Furthermore, both the relatively prolonged incubation period of the disease (the median incubation period was estimated to be 5.1 days, 95% CI 4.5-5.8 [5] or up to 14 days for some cases [6,7] before any symptoms could even be detected) and the postinfection period make it challenging for medical staff to recognize the existence of COVID-19 infections, which could increase the transmission of the disease during these lay periods. Therefore, patients infected with COVID-19, without showing symptoms, are of a great threat to dentists and other members of the dental team. Dentists, thereby, should entertain a high level of awareness and integrity to deal with the disease and be able to control and manage its spread.

There are practical guidelines recommended for dentists and dental staff by the Centers for Disease Control and Prevention (CDC), the American Dental Association (ADA), and the World Health Organization to control the spread of COVID-19 [8-10]. Like with other contagious infections, these recommendations include personal protective equipment, hand washing, detailed patient evaluation, rubber dam isolation, antiretraction handpiece, mouth rinsing before dental procedures, and disinfection of the clinic. In addition, some guidelines and reports have provided useful information about the signs and symptoms of the disease, ways of transmission, and referral mechanisms to increase dentists’ knowledge and prevention practices, so they could contribute, at a population level, in disease control and prevention [1,8].

### Objectives

Despite the availability of prevention guidelines and recommendations on disease control, many dental practices lack the minimum requirements of infection control, which resulted from the low interest in taking the mandatory precautions. This lack of interest in making an extra, but essential, effort could be attributed to the high volume of patients treated in clinics that charge low or reduced dental fees [11,12]. This situation is true for many settings, including some dental clinics in Jordan, which, like many other countries, has a wide range of dental facilities from clinics that properly apply infection control measures to clinics that poorly apply prevention measures. It is important to implement sound prevention measures in dental clinics and to increase the level of awareness among dentists to improve their prevention. Hence, this study aimed to assess the level of awareness, perception, and attitude regarding COVID-19 and infection control among Jordanian dentists.

## Methods

### Study Population

Our study population consisted of dentists who work in Jordan, regardless of their place of work, in either private clinics, hospitals, or health centers. This survey was conducted in March 2020. An online questionnaire using Google Forms was used to collect the data. The sample of dentists was selected through Facebook groups for dentists. These groups were created by members of the Jordan Dental Association, and only dentists who work in Jordan can be involved in these groups by confirming their registration with the Jordanian dental association and their places of work. Although there were numerous groups, only five groups were randomly chosen: Jordanian dentists, dentists without borders, Jordanian dental club, Jordanian society of pediatric dentistry, and Jordanian dentists’ forum. Within the five selected groups, 700 dentists were randomly selected to participate in the study by their Facebook profiles. However, each participant who was randomly selected was contacted individually to make sure that they were a dentist and worked in Jordan. The questionnaires were anonymous to maintain the privacy and confidentiality of all information collected in the study. Ethical approval was obtained from the Institutional Review Board at Jordan University of Science and Technology.

### Study Instrument

The questions on the survey were developed after reviewing pertinent literature and the international guidelines [1,8-10]. The questionnaire was designed in English and comprised of a series of questions pertaining to sociodemographic characteristics, the knowledge of dentists, and their attitudes and perceptions toward COVID-19 and infection control in dental clinics. The survey was a structured multiple-choice questionnaire divided into sections: dentists’ demographic and profession-related characteristics; dentists’ awareness of incubation period, the symptoms of the disease, the mode of transmission of COVID-19, and infection control measures for preventing COVID-19; and dentists’ attitude toward treating patients with COVID-19.

### Data Analysis

Data were analyzed using SPSS (IBM Corp). Descriptive statistical analysis was used to describe items included in the survey. Means and standard deviations were used to describe the continuous variables, and percentages were used to describe the categorical data.

## Results

### Participants’ Characteristics

This study included a total of 368 (245 females and 123 males) dentists, forming a response rate of about 52.6% (386 participated out of 700 invited dentists). Their age ranged from 22-73 years with a mean of 32.9 (SD 10.6) years. Years of dental practice ranged from 1-30 years with a mean of 9.4 (SD 8.9) years. The participants’ characteristics are shown in Table 1. A total of 112 (30.4%) had completed a master or residency program in dentistry, 195 (53.0%) had received training in infection control in dentistry, and 28 (7.6%) had attended training or received lectures regarding COVID-19.

**Table 1:** The characteristics of the 368 dentists enrolled in the study.

Variable		Dentists, n (%)
**Gender**		
	Female	245 (66.6)
	Male	123 (33.4)
**Age (years)**		
	<30	199 (54.1)
	≥30	169 (45.9)
**Years of practice**		
	<5	185 (50.3)
	5-10	59 (16.0)
	>10	124 (33.7)
**Region**		
	Middle	190 (51.6)
	North	148 (40.2)
	South	30 (8.2)
**Health sector**		
	University clinics	112 (30.4)
	Military sector	28 (7.6)
	Private sector	144 (39.1)
	Public sector	84 (22.8)

### Awareness About the Incubation Period, Symptoms, and Mode of Transmission of the COVID-19 Infection

When asked about the incubation period, over one-third of dentists correctly reported 1-14 days. The percentage of dentists who reported the different symptoms of the COVID-19 infection are shown in Table 2. The majority reported fever and cough as symptoms. Diarrhea, vomiting, and runny nose were reported by almost one-third of dentists. Joint and muscle pain was reported by only a few dentists. Over one-third of the dentists reported that patients with COVID-19 infection may present with no symptoms. When they were asked about aspects that should be considered to identify patients at risk of having COVID-19, 316 (85.9%) mentioned the presence of symptoms of a respiratory infection, 347 (94.3%) mentioned history of travel to areas experiencing transmission of COVID-19, and 345 (93.8%) mentioned history of contact with possible infected patients. In addition, most dentists correctly reported known modes of transmission (Table 2).

**Table 2:** Dentists’ awareness about incubation period, symptoms, and mode of transmission of the coronavirus disease infection (N=368).

Variable		Dentists, n (%)
**Incubation period (days)**		
	1-14	133 (36.1)
	2-7	12 (3.3)
	7-14	162 (44.0)
	7-21 days	61 (16.6)
**Symptoms of the COVID-19^a^ infection**		
	Fever	363 (98.6)
	Cough	335 (91.0)
	Shortness of breath	316 (85.9)
	Diarrhea	147 (39.9)
	Vomiting	119 (32.3)
	Runny nose	133 (36.1)
	Sore throat	105 (28.5)
	Red eyes	28 (7.6)
	Skin rash	21 (5.7)
	Joint or muscle pain	7 (1.9)
	May present with no symptoms	127 (34.5)
**Mode of transmission**		
	Coughing and sneezing	333 (90.5)
	Hand shaking	315 (85.6)
	Touching surfaces such as doorknobs and tables	343 (93.2)

^a^COVID-19: coronavirus disease.

### Awareness of Measures for Preventing COVID-19 Transmission in Dental Clinics

The majority of the 368 dentists reported that cleaning hands frequently by using alcohol-based hand rub or soap and water, routinely cleaning and disinfecting surfaces in contact with known or suspected patients, and wearing personal protective equipment can help prevent transmission from patients with known or suspected COVID-19. The percentages of dentists who reported other specific measures are shown in Table 3. Almost all dentists (n=359, 97.6%) reported that it is important to change both masks and gloves regularly to decrease the possibility of transmitting infections to patients and to themselves.

**Table 3:** Dentists’ awareness of measures for the prevention of coronavirus disease transmission in dental clinics (N=368).

Measures for prevention	Dentists, n (%)
Frequently clean hands by using alcohol-based hand rub or soap and water	354 (96.2)
Routinely clean and disinfect surfaces in contact with known or suspected patients	347 (94.3)
Personal protective equipment such as dental goggles, masks, and gloves	342 (92.9)
Put facemask on known or suspected patients	325 (88.3)
Avoid moving and transporting patients out of their area unless necessary	310 (84.2)
All health staff members wear protective clothing	304 (82.6)
Place known or suspected patients in adequately ventilated single rooms	284 (77.2)

### Perception of COVID-19

A total of 65 (17.7%) of the 368 dentists perceived COVID-19 as very dangerous, 264 (71.7%) perceived it as moderately dangerous, and 35 (9.5%) perceived it as not dangerous. Almost one-third (n=135, 36.7%) of dentists believed that COVID-19 is not a serious public health issue. The majority (n=360, 97.8%) reported that it is important to educate people about COVID-19 to prevent the spread of the disease.

### Attitude Toward Treatment of Patients With COVID-19

More than half (n=203, 55.2%) of the 368 dentists reported that COVID-19 symptoms often resolve with time and do not require any special treatment. Regarding dentists’ precautionary actions in the dental clinic, a total of 275 (74.7%) believed that it was necessary to ask patients to sit far from each other, wear masks while in the waiting room, and wash hands before getting in the dental chair to decrease disease transmission, while 80 (21.7%) believed that this was not necessary and could cause panic. However, a total of 304 (82.6%) dentists reported that they prefer to avoid working with a patient with a suspected case of COVID-19.

Dentists reported different attitudes toward a patient sneezing or coughing in their clinics: 161 (43.8%) mentioned that they would refer the patient to the hospital without treating them, 17 (4.6%) mentioned that they would refuse treating the patient and ask them to leave the clinic, 182 (49.5%) mentioned that they would treat the patient and ask them to go to the hospital.

Moreover, a total of 119 (32.3%) dentists reported that they would allow any of their dental staff to work with patients if they had flu-like symptoms. Only 214 (58.2%) reported that they know whom to contact in a situation where there has been an unprotected exposure to a patient with known or suspected COVID-19, and 279 (75.8%) reported that they know what to do if they have signs or symptoms suspected of COVID-19 infection.

For the dentists’ role in spreading information and increasing awareness, a total of 249 (67.7%) dentists reported that the dentist role in teaching others about COVID-19 is very significant, and 94 (25.5%) reported that it is moderately significant.

## Discussion

This survey provides an insight on the level of awareness, perception, and attitude of Jordanian dentists on infection control with a special emphasis on COVID-19 at the time of the outbreak in 2020. This study included a sample of Jordanian dentists. Females were predominant in this sample, which might be explained because the number of female dentists in Jordan is higher than the number of male dentists based on the latest Jordan Dental Association statistics [13].

The estimated incubation period of COVID-19 is up to 14 days [6,7]. Dentists in this study varied in their knowledge about the incubation period of the disease, but it is essential to know the right incubation period because of its role in determining the safe period to treat suspected patients [14]. However, it’s imperative for dentists to carry on with preventive measures for all their patients, all the time. Knowledge about respiratory disease contagion was noticed in other studies to be lower among dentists [15] than among other health care providers [16], despite the proximity of patient to provider present in dental care [4]. Nonetheless, Jordanian dentists in this sample could identify the main symptoms of COVID-19, which helps dentists to recognize the threat and take the necessary actions and is considered essential in the management [14] and control of the spread of the disease [1]. Dentists response to prevention measures were better for personal protective equipment and disinfection and sanitation procedures than for measures applied to dental staff or patients, such as special clothing or ventilation. The latest precautionary actions could possibly be viewed by dentists as extra protective measures that are not necessary when combined with their understanding that infections occur mainly through direct contact between mucous membranes and contaminated hands [9].

There has been no evidence-based specific treatment for COVID-19, and management of COVID-19 has been largely supportive [8]. The current approach to COVID-19 is to control the source of infection; use infection prevention and control measures to lower the risk of transmission; and provide early diagnosis, isolation, and supportive care for affected patients [17]. This fact was reflected by the response of participants to treatment; almost half of dentists thought that the disease self-resolves over time with no need for special treatment. This perception about the disease self-resolution resulted in most participants perceiving COVID-19 as moderately dangerous (n=264/368, 71.7%), and almost one-third believed that COVID-19 was not a serious public health issue. Although their perception about the disease self-resolution could have been explained by their perception about its threat; there were no “local” cases in Jordan at the time of data collection. In addition, dentists’ perception about the seriousness of the disease could be because some (n=80, 21.7%) did not see a need to ask patients to sit far from each other, wear masks while in the waiting room, or wash hands before getting in the dental chair to decrease disease transmission. However, the vast majority (n=304, 82.6%) would prefer to avoid working with a patient with suspected COVID-19 because of the possibility of disease transmission during incubation periods, during which no symptoms may appear [1].

The attitude of dentists regarding what to do in case a patient was sneezing or coughing in their clinics varied; 43.8% (n=161) would refer the patient to the hospital without treating them, 4.6% (n=17) would refuse treatment, and 49.5% (n=182) would treat the patient and then refer them to the hospital. Some dentists (n=119, 32.3%) would allow their dental staff to work with patients if they had flu-like symptoms. During the outbreak of COVID-19, dentists should evaluate risk of transmission through measurements of the temperature of every staff and patient as a routine procedure. Patients should be asked about their health status and any history of recent contact or travel [8]; patients and their accompanying persons should be provided with medical masks upon entry to the clinic. Patients with a fever should be registered and referred to designated hospitals. If a patient has been to any epidemic regions within the past 14 days, quarantining for at least 14 days is recommended. In areas where COVID-19 spreads, nonurgent dental treatment should be postponed [18]. It is still not known when treatments can be done.

Over half of the dentists (n=214, 58.2%) knew whom to contact in a situation of an unprotected exposure to a known or suspected COVID-19 patient, and 75.8% (n=279) reported that they knew what to do if they had signs or symptoms of a suspected COVID-19 infection. By now, there has been no consensus on provision of dental treatment during the COVID-19 epidemic. Based on relevant guidelines and research, dentists should take strict personal protection measures and avoid or minimize operations that may produce droplets or aerosols [18]. A 4-handed technique is useful for infection control, and use of saliva ejectors with low or high volume reduces droplet and aerosol production [1,9]. The consensus of the vast majority (n=360, 97.8%) of dentists about the importance of educating others about COVID-19 to prevent the spread of the disease was high, but they should follow the guidelines from the CDC and ADA and recommendations for infection prevention and control based on the local epidemic situation.

Despite the findings introduced here, it is important to stress that this survey had limitations, including the relatively low response rate, which resulted in a smaller than expected sample size. This could have been caused by the short period of data collection. However, this is considered a moderate sample size. Moreover, this pandemic has caused many to be busy with watching the news and taking care of personal affairs. This means that those who were active on social media during the short period of data collection were the only ones that had the chance to participate in the study. This could result in selection bias and sampling error, which prevents the ability to generalize our results.

In conclusion, Jordanian dentists were aware of COVID-19 symptoms, mode of transmission, infection control, and measures in dental clinics. However, dentists had limited comprehension of the extra precautionary measures that protect the dental staff and other patients from COVID-19. Guidelines released by reputable institutions should be sent by the regional and national dental associations to all registered dentists during a crisis, including this COVID -19 pandemic, to make sure that dentists are well informed and aware of the best practices and recommended disease management approaches.

## Abbreviations

ADA: American Dental Association
CDC: Centers for Disease Control and Prevention
COVID-19: coronavirus disease
